# Facial Pain: RCT between Conventional Treatment and Fascial Manipulation^®^ for Temporomandibular Disorders

**DOI:** 10.3390/bioengineering9070279

**Published:** 2022-06-27

**Authors:** Florence Sekito, Marco Pintucci, Carmelo Pirri, Mariana Ribeiro de Moraes Rego, Mayra Cardoso, Kenia Soares Paixão, Valquiria Ribeiro da Silva, Antonio Stecco

**Affiliations:** 1 Department of Prosthodontic, State University of Rio de Janeiro, Rio de Janeiro 20550-013, Brazil; fmsekito@gmail.com (F.S.); marco.pintucci@gmail.com (M.P.); marianarmrego@gmail.com (M.R.d.M.R.); mayracardoso.mc@gmail.com (M.C.); kenyaspaixao@gmail.com (K.S.P.); valquiria.integra@gmail.com (V.R.d.S.); 2 Department of Neurosciences, Institute of Human Anatomy, University of Padova, 35121 Padova, Italy; 3 Rusk Rehabilitation, New York University School of Medicine, New York, NY 10016, USA; antonio.stecco@nyulangone.org

**Keywords:** Fascial Manipulation^®^, odontology, jaw pain, TMJ disorders, facial pain, rehabilitation

## Abstract

Background: To investigate the effectiveness of a specific manual therapy, Fascial Manipulation^®^ (FM), in comparison with conventional treatments in temporomandibular disorders (TMD) patients using a two-arm randomized controlled trial. Methods: The study consisted of 28 patients that were divided in two groups (Group 1: Fascial Manipulation^®^ vs. Group 2: conventional TMD treatment). The Verbal Rating Scale (VRS), RDC/TMD, electromyography (EMG) and Pression/Pain Evaluation on Masseter and Temporalis Muscle were assessed with different times. Results: In both groups, the improvement in pain was evident on the VRS scale (*p* < 0.0001) and pain-free opening (*p* < 0.001). In Group 1, the recovery of the function was faster; maximum unassisted opening T0 vs. T1 (*p* = 0.001). Conclusions: FM^®^ can be used as an effective method for facial pain, being a rapid, safe and cost-effective approach to reduce pain, gain function and mouth opening that can be used prior to occlusion stabilization appliances.

## 1. Introduction

Nowadays, the prevalence of orofacial pain is between 3% and 12% and at least twice as prevalent in women as men [1]. It is defined as a heterogeneous group of musculoskeletal and neuromuscular conditions involving the temporomandibular joint complex, surrounding musculature and osseous components [2]. Among these, temporomandibular disorders (TMDs) are the most prevalent and debilitating conditions, involving the head and face, with pain affecting the jaw, ears, eyes and frequently causing headache and neck pain [3]. The etiology of chronic TMD is multifactorial and includes structural, functional, environmental, social and psychological factors [4]. The symptomatology includes myalgia, usually presenting as a dull, aching pain due to continued muscle tension. Myofascial pain (MFP) also presents as a dull, continuous aching pain that varies in intensity. MFP produces pain upon palpation that is usually local but may refer to other sites, as mapped out by Simons [5]. MFP tends to be seen in muscle pain conditions of a more chronic nature, in which the tension is unremitting. Trigger points can often be seen in MFP and may be localized to a taut band of muscle. However, the multifactorial pathophysiology of TMJ-related pain is far from being completely understood and effective management of pain has not been established yet [6]. In addition to pain, which may be located on the head, neck and face, symptoms of TMD may include clicks, crackles and/or tinnitus [7].

In the literature, the conventional treatments for TMD include patient education, home-care programs, physical therapy, musculoskeletal manual approach, pharmacotherapy, nonsteroidal anti-inflammatory drugs (NSAIDs), local anesthetics, intracapsular injection of corticosteroids, muscle relaxants, antidepressants, occlusal appliance therapy and occlusal adjustment [8]. Surgical care is only indicated when non-surgical therapy has been ineffective [9]. Regarding conservative, non-medical and non-dental treatments, the musculoskeletal manual approaches are noted for their impact on biological tissues. It involves a reduction in biomechanical and neurophysiological imbalances [10] responsible for pain relief, reduction in muscle activity [11] and improving function [12]. Unfortunately, despite the evidence seen in two systematic reviews that support manual therapy as producing favorable outcomes in TMD [13,14,15], the real effectiveness of different types of manual therapy in TMD remains unclear. Currently, evidence suggests that one type of manual therapy, the Fascial Manipulation^®^ (FM), is an effective technique in the treatment of musculoskeletal pain, in a variety of movement disorders, such as spine, head, and upper and lower limbs [16,17,18,19]. Thus, considering this context, the aim of this random controlled trial (RCT) was to analyze the effectiveness of FM^®^ in comparison with conventional treatments in TMD patients.

## 2. Materials and Methods

### 2.1. Study Design and Participants

This study was a 2-arm randomized controlled trial and was approved by the Research Ethics Committee, National Health Council Resolution, Brazil (No. 196/96) and registered in the Registro Brazileiro de Ensaios Clínicos (ReBEC) Plataforma Brazil”: http://plataformaBrazil.saude.gov.br/login.jsf, accessed on 6 June 2018, under protocol number: 84880217.0.0000.5259. All participants gave their signed informed consent to be in the study. The enrolment was made by the Dentistry Faculty TMD clinic of the Universidade do Estato do Rio de Janeiro (Brazil). Screening for inclusion was performed by a unique examiner, trained and calibrated according to specifications established by the International Research Diagnostic Criteria for TMD (RDC/TMD) Consortium. The inclusion criterion was positivity to RDC/TMD AXIS I at the clinical examination, lasting more than 4 months. All subjects included had diagnosis of myo-fascial pain, with or without limited opening. Subjects presenting disc displacement with and without reduction were included. It has to be noted that someone presented intraarticular noise such as clicking or crepitus. Sample size was calculated following a previous study [19].

The exclusion criteria were: diagnosis of osteoarthritis (III b) and osteoarthrosis (III c) according to the RDC/TMD, psychiatric conditions, chemical dependence, fibromyalgia, rheumatologic diseases, neurological disorders, neuropathies and polytraumatized patients. The patients were randomly allocated into 2 groups using block randomization and opaque envelopes to conceal the allocation. Neither the investigators nor the patients could know to what group each patient would be allocated. Group assignment was performed following the initial evaluation but prior to the initial treatment session.

### 2.2. Procedures and Data Collection

#### 2.2.1. Group 1 

**Figure 1:**
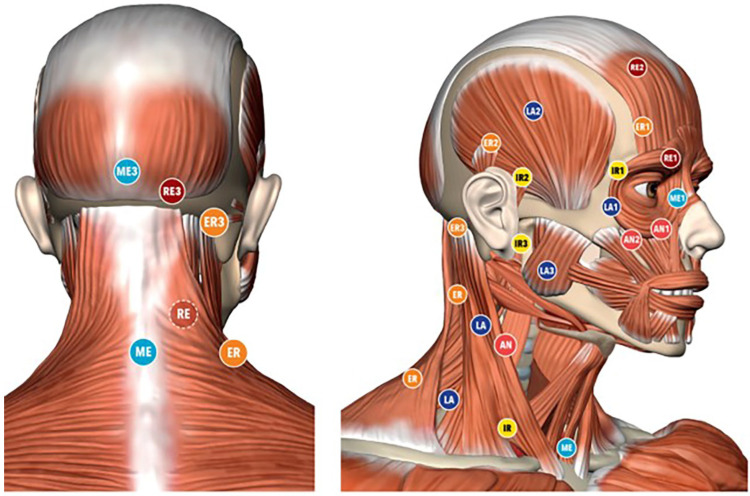
Location of the Center of Coordination points: IR: intra rotation; ER: extra rotation; ME: medio motion; LA: latero motion; AN: ante motion; RE: retro motion.

The subjects in the Fascial Manipulation^®^ (FM) group (Group 1) underwent five FM treatments weekly (about 45 min, 1 h each). The therapists used their elbow and knuckles, generating a deep friction for 3–5 min over each point. The points were selected after a specific assessment process, guided by a specific chart (FM^®^ chart) [19], involving medical records, clinical examination of specific movements and palpatory verifications. The latest includes patient pain rate, radiation and, most importantly, the presence of tissue stiffness, termed “densification” [20]. During the clinical history, dysfunctional segments were identified with an emphasis on the chronology. This chronology-based evaluation leads to hypothesis development of the patient’s current symptomatology cause based on previous musculoskeletal events, which may have been causing compensations. The treatment was applied over specific points, called Centers of Coordination (CCs) and Centers of Fusion (CFs), that are anatomically safe because they do not overlie major superficial nerves and veins (Figure 1). Additional guidance for point selection includes avoiding the patients’ excessively painful areas, where inflammation, lesions or even fractures could be present. In each session a total 5–7 CCs plus CFs were treated. According to the literature, authors consider the application of manual therapy to the cervical and upper thoracic area relevant [21] due to the neuroanatomical connection between these two segments and also due to the biomechanical relationship between the cervical and orofacial regions [22]. Following this, it was decided to apply FM in the head, neck and upper part of the chest regions where previous studies were already conducted with this technique [18,23].

#### 2.2.2. Group 2

The subjects in Group 2 received temporomandibular disorder treatment (TMDT) which consisted of: Michigan Occlusal Appliance, anesthetic injection and dry needling of muscle trigger points. Anesthetic injection was performed with Lidocaine 0.5% used across all patients with a range of 0.3–0.8 mL for point [24]. The skin was cleaned with antiseptic swabs and the injections were administered using sterile aseptic technique by the same operator. Surface anatomical landmarks were used to locate the muscles injected and precautions were taken to avoid intravascular injection. During each session, injections were performed over 8 points on the masticatory muscles that presented positive trigger points: the trapezius at the level of the omohyoid, temporalis, masseter, splenius capitis and levator scapula muscles. Patient received anesthetic injection and dry needling for 3 weekly sessions each. Patients in the TMDT group were also treated with oral appliances (OAs) (also known as flat plane stabilization appliance, Michigan splint, muscle relaxation appliance or gnathologic splint) fabricated for the maxillary arch. OAs are processed acrylic devices that have been used for the management of TMD for years, with different designs. Studies have reported a reduction in TMD symptoms or, at least, sufficient evidence to justify their use for myalgia and arthralgia of the masticatory system [25]. In an extensive review on the use of OAs and the management of TMD, it was concluded that OAs are still regarded as a useful adjunct therapy for some TMD cases [26]. Splints were made individually through a specific methodology of fabrication. A method of direct application of acrylic composite was used. Splints were individually adjusted each week for a total of 5 weeks. Patients were instructed to wear the splint every night and for up to three non-continuous hours during the day if the pain intensity was high. After the 5 weeks of splint adjustment, patients were instructed to continue the use of the appliance only at night until the 6-month follow-up (Table 1).

### 2.3. Data Collection

**Figure 2:**
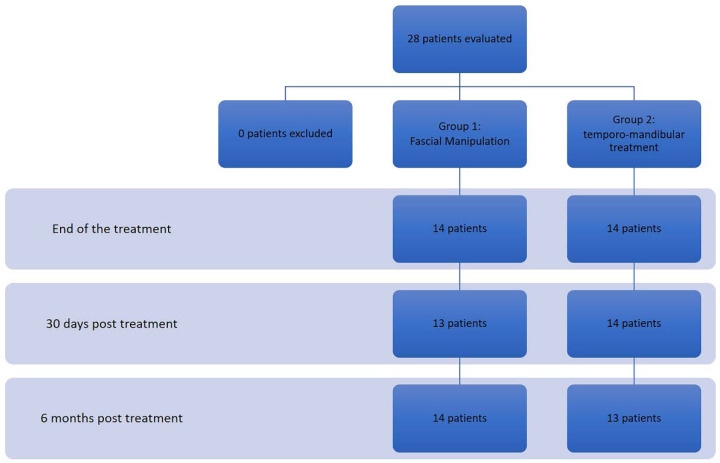
Flow chart of the research.

The following outcome measurements were evaluated: the Verbal Rating Scale (VRS), RDC, electromyography (EMG) evaluation at Maximum Voluntary Isometric Contraction (MVIC) (isometric clenching) on Masseter and Temporalis Muscle. All the evaluations were performed before treatment initiation (T0), at treatment end (T1) and at 30 days (T2) and 6 months (T3) after the interventions (Figure 2). The clinical staff was aware and available to assist any patients with the formalities of this trial. The researchers, involved in the treatments, were blind to all the data until the end of the trial

#### 2.3.1. VRS

VRS was used to quantify the intensity of pain at rest, using adjectives to describe different levels of pain. Each subject was asked to select the adjective which fit best to the pain intensity.

#### 2.3.2. RDC/TMD

At the initial examination, the occurrence of TMJ clicking, crepitus, or jaw opening interferences with or without pain were recorded. Light but steady force was applied with fingertips over the lateral and posterior aspects of the TMJs when the mandible was at rest/closed position and at open position. The clinician viewed the patient’s opening and closing patterns to note any mandibular deviations. The evaluation of mandibular ROM consisted of measuring comfort opening, active opening, passive opening, protrusion, and left and right lateral excursions with a millimeter ruler while noting the pain severity and location with jaw movement. Comfort opening was determined by the patient opening as wide as possible without any pain. Active opening was determined by the patient opening as wide as possible with pain. Passive opening was determined by gently stretching patient’s dental arches from the clinician, who was also noting any soft or hard end feel. The amplitude should, presumably, exceed the active opening.

#### 2.3.3. EMG

EMG is a low-cost, non-invasive bioelectrical instrument that allows assessment of muscular electrical activity and can be used both in clinical settings and in the diagnosis of myogenic TMD [27], as well as in research for diagnosis and, further, to quantify the effect of treatments on the TMD population [28]. The participants were subjected to EMG evaluations using the New miotool (Miotec Equipamentos biomédicos ltda, Petrópolis, Porto Alegre, RS, Brazil) with 14-bit resolution and a sampling frequency of 2000 Hz, IRMC > 126 dB and signal noise rate < 2 LSB, security insulation 3000 V (rms). Simple, differential, active electrodes were used for the capture of the action potential in the right and left masseter and temporal muscles. The sensor has a pre-amplifying circuit with an automatic gain. Input impedance 10 Ohm was used with a self-adhesive electrode (Medtrace, Kendall™/Covidien Medi-Trace® Series Electrode, Brooklin São Paulo, SP Brazil). Butterworth filter with band-pass 20–500 Hz was used and skin was cleaned with cotton soaked in a 70% alcohol solution. The electrodes were then positioned over the right and left masseter and temporal muscles. A reference electrode was attached for the styloid process. During the examination, the volunteers remained in orthostatic position, with erect trunk, Frankfurt plane parallel to the ground (without flexion and extension of the neck) and eyes open. Electromyography signal recordings were made, with a duration of 10 s, during maximum voluntary contraction (jaw clenching). All procedures were performed three times, with a thirty-second interval between isometric contractions to avoid muscle fatigue. After electromyography signal acquisition, all the data were processed in Miotec Suite (Miotec Equipamentos biomédicos ltda, Petrópolis, Porto Alegre, RS, Brazil) to analyze the root square mean (RMS) in μV.

#### 2.3.4. Pression/Pain Evaluation

Each volunteer was subjected to a compression of 1 N/m in the anterior, medial and inferior part of the temporal and masseter muscle bilaterally. Patients were asked to rate from 0 (no pain) to 4 (excruciating pain) the intensity of the pain reported on the sites while compression was generated with an analogic algometer. Volunteers were seated in a relaxed position during the entire evaluation process.

### 2.4. Statistical Analysis

Statistical analysis was performed using GraphPad PRISM 8.4.2 (GraphPad Software Inc., San Diego, CA, USA), and *p* < 0.05 was always considered as the limit for statistical significance. The normality assessment was carried out using the Kolgomorov–Smirnov test. Descriptive statistics were calculated for both groups separately, including measures of central tendency and their dispersion ranges using the mean and standard deviation (SD) to describe parametric data and the median and interquartile range (IR) to describe non-parametric data. Student t-test was used to evaluate group differences in demographics. Difference between two groups at the different times (T0, T1, T2 and T3) about the continuous variables were statistically analyzed by 3-way analysis of variance (ANOVA) mixed model followed by Dunnett’s multiple comparisons test for multiple comparisons. Some continuous variables at the different times (T0, T1, T2 and T3) were statistically analyzed by 2-way analysis of variance (ANOVA) mixed model followed by Tukey’s multiple comparisons test for multiple comparisons. Chi-square test for trend was calculated for categorial variables.

## 3. Results

The study included 28 patients, aged 23–65 (45.28 ± 13.75). Statistical analysis confirmed the homogeneity of the two groups regarding age (group 1 = 46.25 ± 13.12 years old; group 2 = 49.33 ± 13.64 years old), sex (only male), VRS score (group 1 = 9.33 ± 0.89; group 2 = 8.83 ± 1.34), mouth opening without pain (group 1 = 45.31 ± 11.40 mm; group 2 = 46.05 ± 8.23 mm) and maximum opening with assistance (group 1 = 49.24 ± 11.56 mm; group 2 = 49.52 ± 6.55 mm). It was decided to retain the null hypothesis because the samples were mostly homogenous and so were comparable in most of the aspects. Non-parametric tests were still used due to the small number in each single group.

**Figure 3:**
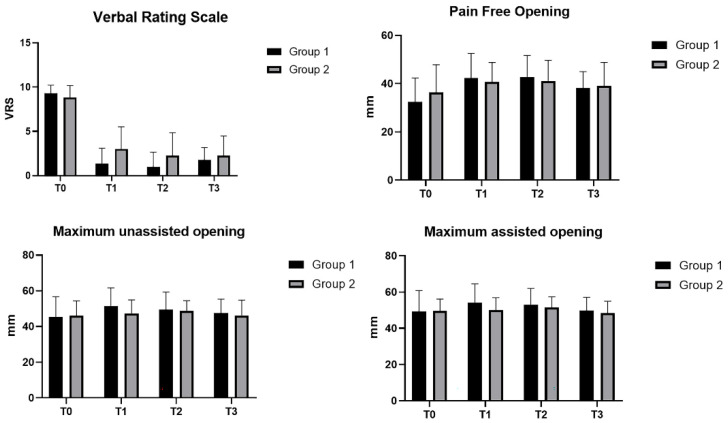
Verbal rating scale; pain-free opening; maximum unassisted opening; maximum assisted opening. T0 = before treatment; T1 = after treatment; T2 = 30 days; T3 = 6 months; Group 1: Fascial Manipulation; Group 2: temporomandibular disorder treatment.

In both groups, improvement in pain, seen via the VRS scale, between initial condition (T0) and after the treatment (T1), was maintained at 30-day follow-up (T2) and 6-month follow-up (T3) (*p* < 0.0001) (Figure 3). No statistically significant differences were present between the two groups (*p* > 0.05).

Regarding Table 2, statistically significant differences (*p* < 0.001) were found in both group between T0 and the other times (T1; T2; T3).

The patients passed from the symptomatic condition with the following VRS data (Group 1: 9.33; Group 2: 8.87) to asymptomatic (Group 1: 1.33; Group 2: 2.62) after the treatment. At 30 days, the pain was 1.16 in Group 1 and 2.25 in Group 2. At 6 months, the pain reported by patients in Group 1 had a mean value of 2.08, while in Group 2: 1.56. The difference in the pain-free opening in both groups was statistically significant among different times (T0, T1, T2 and T2) (*p* < 0.001) (Figure 3). No statistically significant differences were present between the two groups (*p* > 0.05). Regarding Table 3, statistically significant differences (*p* < 0.001) were found only in the first group, in T0 vs. T1 (*p* = 0.0026) and T0 vs. T2 (*p* = 0.0149).

Maximum unassisted opening improved in both groups in the times (*p* = 0.0025) (Figure 3). No statistically significant differences were present between the two groups (*p* > 0.05). Regarding Table 4, a unique statistical difference was found in Group 1 between T0 vs. T1 (*p* = 0.0010). Maximum assisted opening improved in both groups in the times (*p* = 0.0013) (Figure 3). No statistically significant differences were present between the two groups (*p* > 0.05).

Regarding Table 5, in Group 1, the difference between T0 and T1 was statistically significant (*p* = 0.0031), whereas in Group 2, among T0 vs. T2 (*p* = 0.0281) and T2 vs. T3 (*p* = 0.0144). No statistically significant differences were present between the two groups (*p* > 0.05).

**Figure 4:**
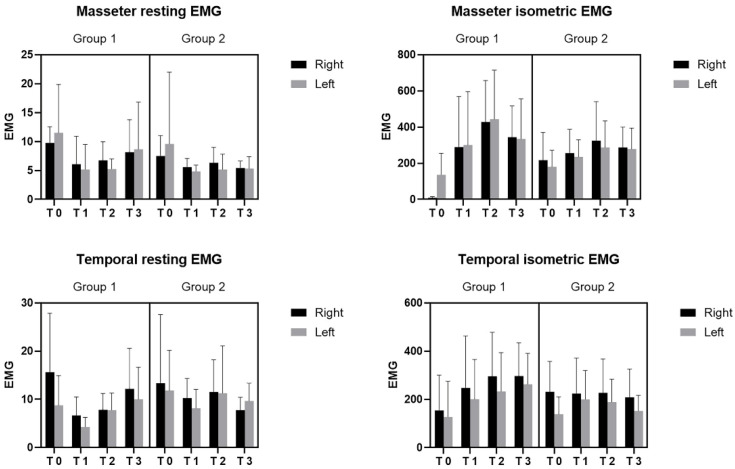
EMG evaluation in masseter and temporal muscles. T0 = before treatment; T1 = after treatment; T2 = 30 days; T3 = 6 months; Group 1: Fascial Manipulation; Group 2: temporomandibular disorder treatment.

The difference among EMG evaluations in masseter and temporal muscles is reported at resting position and at isometric contraction in Figure 4.

At resting position, for the masseter muscle, no statistically significant differences were present between the two groups (*p* > 0.05), whereas it was statistically different at different times within each group (*p* = 0.0004). At isometric contraction, for the masseter muscle, statistically differences were found among various times (*p* < 0.0001) and between the two groups (*p* = 0.0064). At resting position, regarding the temporal muscle, no statistically significant differences were present between the two groups (*p* > 0.05), whereas it was statistically different at different times (*p* = 0.0081). At isometric contraction, for the temporal muscle, statistical differences were found among various times within each group (*p* = 0.0461).

The difference regarding palpation in the origin of the masseter muscle was statistically significant among the various times (*p* < 0.0001) for both groups. Moreover, for the body of the masseter muscle, the differences were statistically significant among the various times (*p* < 0.0001) for both groups, between the two groups (*p* = 0.0011) and between right and left in both groups (*p* = 0.0011). For the palpation of the insertion of the masseter muscle, the differences were statistically significant among the various times (*p* < 0.0001) for both groups and between the two groups (*p* = 0.0074) (Figure 4).

**Figure 5:**
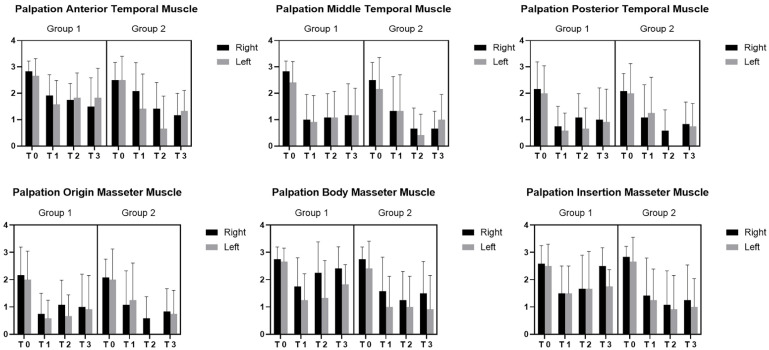
Palpation of the temporal and masseter muscles. T0 = before treatment; T1 = after treatment; T2 = 30 days; T3 = 6 months; Group 1: Fascial Manipulation; Group 2: temporomandibular disorder treatment.

The difference regarding palpation of the anterior temporal muscle was statistically significant among the various times (*p* < 0.0001) for both groups. Moreover, a statistically significant difference between the groups was found (*p* = 0.0087). For the middle temporal muscle, it was statistically significant among the various times (*p* < 0.0001) for both groups. No statistically significant differences were present between the two groups (*p* > 0.05). In terms of the posterior temporal muscle, it was statistically significant among the various times (*p* < 0.0001) for both groups (Figure 5). No statistically significant differences were present between the two groups (*p* > 0.05).

**Figure 6:**
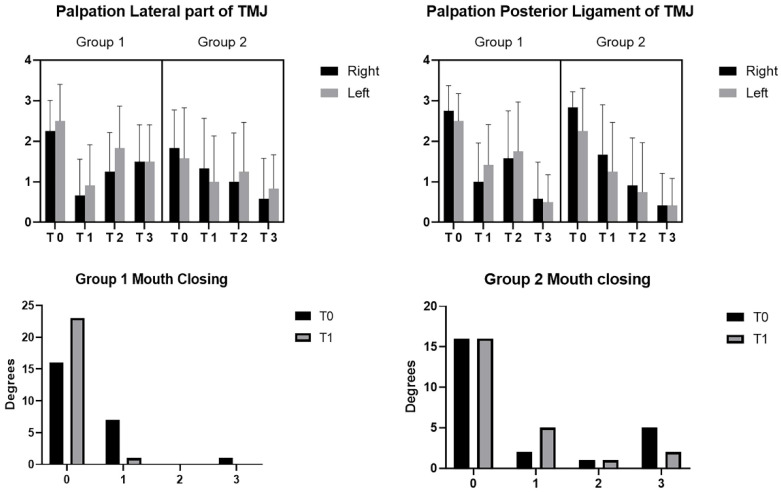
Palpation in lateral part of TMJ and posterior ligament of TMJ: T0 = before treatment; T1 = after treatment; T2 = 30 days; T3 = 6 months; Group 1: Fascial Manipulation; Group 2: temporomandibular disorder treatment. Joint sound evaluation of the mouth closing: 0 = no pain, 1 = painful, 2 = intense pain, 3 = intense pain with radiation. T0 = Initial measurement; T1 = after treatment.

The differences regarding palpation in the lateral part of the TMJ were statistically significant among the various times (*p* < 0.0001) for both groups and between the two groups (*p* = 0.0117) (Figure 5). For the palpation in the posterior ligament of the TMJ, it was statistically significant among the various times (*p* < 0.0001) for both groups (Figure 6). No statistically significant differences were present between the two groups (*p* > 0.05).

Patients in Group 1 reported a decrease in joint sound only between T0 and T1 (*p* = 0.0180) during mouth closing (Figure 6). No statistical difference was found in the two groups in the other times (*p* > 0.05), and neither difference was found in the two groups in the different times (*p* > 0.05) during mouth opening. No statistically significant differences were present between the two groups (*p* > 0.05).

## 4. Discussion

Both groups received effective treatments that decreased pain, not only after the treatment but also during the following 6 months. The values of VRS at T0, T1, T2 and T3 were the same across group categories, showing how both the therapeutic approaches were valid, even if different times and costs were applied. Group 2 received more expensive treatments in comparison to Group 1, regarding time consumption, cost for the procedures and instruments used. Interestingly, ROM improved after the treatments in Group 1 in the opening at rest, unassisted and assisted motion testing, but it did not last. This could be explained due to the fact that the treatment applied to the patient was brief, being completed in 5 weeks. It was also hypothesized that most of the patients avoided full opening of the mouth due to fear that the pain would recur. Since they did not open their mouths properly for a long time, this limitation could be considered a more psychological than a quantitative limitation [29]. This hypothesis is well supported thanks to the max-opening-without-help improvement obtained at the follow-up by Group 2, which reached a statistical significance only at the first follow-up, as a possible result of the education process that patients went through during the multiple visits for the combined treatments, defined in the Group 2 treatment plan.

The results obtained in the masseter and temporalis showed how the two treatments can decrease muscle sensitivity. Group 1 received treatment that included areas of the shoulder, promoting decreased tension and sensitivity in cranial areas. Following the literature and the guidelines of the method, craniomandibular and neck disabilities have been associated with painful chronic TMD [30]. For this reason, FM guides the therapist through a global approach [18,23], evaluating the myofascial continuity [20] that plays a critical role in the biomechanics of the human body. EMG was able to prove, with objective data, how these therapies were able to restore the normal muscle activation, at least in the post treatment and 30-day follow-up. At the 6-month follow-up, only the right temporalis and masseters muscle in Group 1 significantly improved in isometric contraction compared to T0. Better results were found in Group 1, proving how FM is able to restore and improve myo-electrical activity, which may improve muscle contraction and, possibly, strength. The increase in the microvolt measured supports the hypothesis that both of the treatments were able to increase the number of muscle fibers that patients were able to recruit during active contraction. These results are in conflict with some authors who showed a transient effect, which did not last more than 2 weeks [31]. However, the treatments applied were different. This can explain these major differences. The change in strength can be explained as a modification of the activation of the muscle spindles that modulate the activation of motor units. It has been proven that the muscle spindle capsule is located in a split of the perimysium, a component in the fascial system. Spindle capsules are made up of two separate layers, with hyaluronan inside [32]. Some authors support the idea of the ability of mechanical stress, generated by manual therapy, to change the quality of the hyaluronan and, consequently, the stiffness in the muscle spindle capsule. This change will then modify the function of the entire muscle spindle [33]. The FM treatment was shown to be able to reduce pain as well as the gold standard in orofacial pain management in only five sessions, with a minor investment of time and resources. Even if FM shares some similarities with other techniques, it uses a different rationality and clinical approach. While the deep friction of FM can be compared to other techniques, the reasoning behind the choice of points treated presents major differences. The points are selected after a specific assessment process, involving clinical history taking, a clinical examination of specific movements as well as palpatory verifications [20]. Apart from the use of clinical procedures (palpation, auscultation, measuring of active and passive mandibular mobility), FM requires additional orthopedic tests, implying the need for a modern, biomedical approach, based on knowledge of the human fascial system, but, at the same time, uses an individualized approach to the patient, as recommended by many authors [34].

*Limitations*: Most of the data were homogenous, which allowed the use of parametric statistic tests. However, the decision to use non-parametric testing was made due to the small numbers in the groups. The education levels of the participants were heterogeneous and some communication problems occurred, in particular, during the follow-up.

## 5. Conclusions

FM could be used as an effective method for facial pain, being a rapid, safe and cost-effective approach to reduce pain and gain function and mouth opening that can be used prior to occlusion stabilization appliances. The results confirm the evidence from the scientific literature, suggesting that manual therapy may be useful for TMD patients. Future studies on larger samples are needed in order to facilitate a more targeted approach to treatment.

## Figures and Tables

**Table 1:** The scheme of the two different approaches, with the specific timing, duration, quantity, frequency and number of sessions. h: hours; min: minutes; TMDT: conventional temporomandibular disorder treatment.

Interventions	Fascial Manipulation^®^		TMDT	
	Fascial Manipulation^®^	Michigan Occlusal Appliance	Anesthetic Injection (Lidocaine 0.5%)	Dry Needling of Muscle Trigger Points
Duration	1 h (3–5 min for each point)	Every night + 3 daytime h for 5 weeks. Then only at night for 6 months	0.3–0.8 mL	-
Quantity	6 points	-	8 points	8 points
Frequency	Weekly	Weekly adjustment	Weekly	Weekly
Number of sessions	5	5	3	3

**Table 2:** Comparison between VRS within groups.

	Group 1	Group 2
T0 vs. T1	* <0.0001	* 0.0001
T0 vs. T2	* <0.0001	* <0.0001
T0 vs. T3	* <0.0001	* <0.0001
T1 vs. T2	0.9553	0.8622
T1 vs. T3	0.8245	0.7372
T2 vs. T3	0.5482	>0.9999

T0 = before treatment; T1 = after treatment; T2 = after 30 days; T3 = at 6 months; Group 1: Fascial Manipulation; Group 2: temporomandibular disorder treatment. *: statistically significant *p*-values.

**Table 3:** Pain-free opening.

	Group 1	Group 2
T0 vs. T1	* 0.0026	0.0738
T0 vs. T2	* 0.0149	0.1304
T0 vs. T3	0.1214	0.7188
T1 vs. T2	0.9968	0.9846
T1 vs. T3	0.4949	0.8486
T2 vs. T3	0.283	0.5873

T0 = before treatment; T1 = after treatment; T2 = after 30 days; T3 = at 6 months; Group 1: Fascial Manipulation; Group 2: temporomandibular disorder treatment. *: statistically significant *p*-values.

**Table 4:** Maximal unassisted opening.

	Group 1	Group 2
T0 vs. T1	* 0.001	0.61
T0 vs. T2	0.1349	0.1416
T0 vs. T3	0.7031	0.9995
T1 vs. T2	0.4181	0.825
T1 vs. T3	0.1505	0.9056
T2 vs. T3	0.5879	0.3008

T0 = before treatment; T1 = after treatment; T2 = 30 days; T3 = 6 months; Group 1: Fascial Manipulation; Group 2: temporomandibular disorder treatment. *: statistically significant *p*-values.

**Table 5:** Maximal assisted opening.

	Group 1	Group 2
T0 vs. T1	* 0.0031	0.9605
T0 vs. T2	0.221	* 0.0281
T0 vs. T3	0.9877	0.4979
T1 vs. T2	0.8384	0.5106
T1 vs. T3	0.116	0.4291
T2 vs. T3	0.3314	* 0.0144

T0 = before treatment; T1 = after treatment; T2 = 30 days; T3 = 6 months; Group 1: Fascial Manipulation; Group 2: temporomandibular disorder treatment. *: statistically significant *p*-values.

## Data Availability

The data presented in this study are available on request from the corresponding author.
